# Immunoprofiling of Adult-Derived Human Liver Stem/Progenitor Cells: Impact of Hepatogenic Differentiation and Inflammation

**DOI:** 10.1155/2017/2679518

**Published:** 2017-04-11

**Authors:** Hoda El-Kehdy, Camillo Sargiacomo, Mohammad Fayyad-Kazan, Hussein Fayyad-Kazan, Catherine Lombard, Laurence Lagneaux, Etienne Sokal, Mehdi Najar, Mustapha Najimi

**Affiliations:** ^1^Institut de Recherche Expérimentale and Clinique (IREC), Laboratory of Pediatric Hepatology and Cell Therapy, Université Catholique de Louvain, 1200 Brussels, Belgium; ^2^Institut de Biologie et de Médecine Moléculaires, Université Libre de Bruxelles, 6041 Gosselies, Belgium; ^3^Laboratory of Cancer Biology and Molecular Immunology, Faculty of Sciences I, Lebanese University, Beirut, Lebanon; ^4^Laboratory of Clinical Cell Therapy, Institut Jules Bordet, Université Libre de Bruxelles (ULB), Brussels, Belgium

## Abstract

Adult-derived human liver stem/progenitor cells (ADHLSCs) are, nowadays, developed as therapeutic medicinal product for the treatment of liver defects. In this study, the impact of hepatogenic differentiation and inflammation priming on the ADHLSCs' immune profile was assessed in vitro and compared to that of mature hepatocytes. The constitutive immunological profile of ADHLSCs was greatly different from that of hepatocytes. Differences in the expression of the stromal markers CD90 and CD105, adhesion molecules CD44 and CD49e, immunoregulatory molecules CD73 and HO-1, and NK ligands CD112 and CD155 were noted. While they globally preserved their immunological profile in comparison to undifferentiated counterparts, differentiated ADHLSCs showed a significant downregulation of CD200 expression as in hepatocytes. This was mainly induced by signals issued from EGF and OSM. On the other hand, the impact of inflammation was quite similar for all studied cell populations with an increased expression level of CD54 and CD106 and induction of that of CD40 and CD274. In conclusion, our immune profiling study suggests CD200 as a key factor in regulating the immunobiology of differentiated ADHLSCs. A better understanding of the molecular and physiological events related to such marker could help in designing the optimal conditions for an efficient therapeutic use of ADHLSCs.

## 1. Introduction

To date, cell therapy for metabolic liver diseases and hepatic injuries mainly relies on the use of various types of cells including hepatocytes, liver sinusoidal endothelial cells, mesenchymal stem cells (MSCs), endothelial progenitor cells, and macrophages [1]. However, several limitations and problems are associated with these cells that will finally have a critical impact on the efficiency of liver cell therapy [1]. Adult-derived human liver stem/progenitor cells (ADHLSCs) are obtained, in vitro, after primary culture of healthy adult human liver parenchymal cell fraction [2]. These cells exhibit a fibroblastic morphology and a hepatomesenchymal phenotype [2]. Even though considered as MSC-like, much less is known about ADHLSCs in comparison to the classical MSCs. It is reported that ADHLSCs, in their basal state, demonstrate distinct expression and secretion profiles [3, 4]. When exposed to in vitro hepatogenic differentiation, ADHLSCs are capable to differentiate, either in vitro or in vivo, into hepatocyte-like cells [4]. Recently, upon characterizing the immunological profile of ADHLSCs, our group showed that besides their potency in suppressing T cell proliferation, ADHLSCs are nonimmunogenic since they are negative for HLA-DR as well as for costimulatory molecule expression [5]. Altogether, their self-renewal potential, ability to acquire hepatocyte features, and their hypoimmunogenicity highlight ADHLSCs as a potential alternative cell source for liver cell transplantation. However, achieving these goals involves addressing different aspects related to their safety and efficacy. For instance, tracking the changes of ADHLSCs' immunological profile following hepatogenic differentiation and after exposure to inflammation is missing.

Accordingly, the current work was designed to learn more about the ADHLSC immune profile modulation after in vitro hepatogenic differentiation and in an inflamed environment. Flow cytometry analysis demonstrated the dissimilarity between hepatocytes and undifferentiated ADHLSCs as shown by the expression of stromal markers CD90 and CD105, adhesion molecules CD44 and CD49e, immune regulatory molecules CD73 and HO-1, and NK ligands CD112 and CD155. We also confirm that differentiated ADHLSCs do not acquire a complete and similar hepatocyte immune phenotype but rather maintain a profile comparable to that of undifferentiated cells. However, a specific and major downregulation of CD200 expression was highlighted to reach basal levels as those exhibited by hepatocytes. The impact of inflammation was quite similar for all studied cell populations with an increase in the expression level of CD54 and CD106 and induction of that of CD40 and CD274.

Downregulation of CD200 expression occurred early during the in vitro hepatocytic differentiation process and is modulated by the epidermal growth factor (EGF). Oncostatin M is also inhibiting CD200 mRNA expression at the late maturation phase of the hepatogenic differentiation process. Besides identifying CD200 expression level as an important marker to distinguish hepatic differentiated versus undifferentiated ADHLSCs, our observations suggest a potential role for CD200 in regulating the immunobiology of ADHLSCs. The loss of CD200 might have critical repercussions on the immunobiology of the differentiated ADHLSCs and specifically for liver immunology.

## 2. Materials and Methods

### 2.1. Isolation and Culture of Hepatocytes and ADHLSC

The present study was accepted by the institution ethical review board for the use of human-derived tissue, under appropriate informed consent of tissue donors. An agreement from the Belgian Ministry of Health was delivered for hepatocytes and hepatic stem cell isolation and banking.

Liver cell suspensions were recovered after a two-step collagenase perfusion technique of livers from healthy cadaveric donors. Following filtration and low-speed centrifugation, the parenchymal fraction, predominantly constituted by hepatocytes, was recovered and seeded as primary cultures. ADHLSCs were then obtained as previously described (Najimi et al. [2]). The cells were cultured using DMEM containing 4.5 g/l glucose (Life Technologies) supplemented with 10% fetal calf serum (FCS) (Life Technologies) and 1% penicillin/streptomycin (Life Technologies), at 37°C in a fully humidified atmosphere (5% CO2). When reaching 80% confluence, cells were lifted with 0.05% Trypsin-EDTA (Life Technologies) and seeded at a density of 5000 cells/cm^2^. The viability of recovered cells was evaluated using Trypan blue exclusion assay at each passage.

### 2.2. Hepatogenic Differentiation of ADHLSC

After seeding at 10^4^ cells/cm^2^ in six-well plates coated with rat tail collagen type I, ADHLSCs were maintained in expansion medium for 48 h. Thereafter, cells were sequentially incubated with hepatogenic differentiation cocktail containing specific growth factors and cytokines as previously described (Najimi et al. [2]). Except for step 1 (one medium change), the differentiation medium was changed each 3 days and the cells were microscopically followed at a regular basis. At the end of the maturation step, cells were harvested for analyses to appreciate the level of their phenotypic and functional differentiation. Control undifferentiated cells were kept during the whole process of differentiation in IMDM medium supplemented with 1% FCS.

### 2.3. Inflammation Priming

Adherent cells were primed overnight using a cocktail of proinflammatory cytokines: 25 ng/ml IL-1*β* (Peprotech, Rocky Hill, NJ, USA), 10^3^ U/ml IFN-*γ*, 50 ng/ml TNF-*α*, and 3×10^3^ U/ml IFN-*α* (all from ProSpec Inc., Rehovot, Israel).

### 2.4. Flow Cytometry

A panel of conjugated monoclonal antibodies (Table 1) was used to assess the phenotype of the cells studied under different culture conditions. After labelling, acquired results were analyzed by using a MACSQuant analyzer (Miltenyi Biotec) [5].

### 2.5. RT-qPCR

ADHLSCs' total RNA was extracted using TriPure Isolation Reagent (Roche, Belgium). First-strand cDNA was synthesized using high-capacity cDNA reverse transcription kit according to manufacturer's instructions (Applied Biosystems) and subsequently diluted with nuclease-free water (Invitrogen) to 10 ng/*μ*l cDNA. RT-PCR amplification mixtures (25 *μ*l) containing 25 ng template cDNA, Master Mix buffer (12,5 *μ*l) (Applied Biosystems), and the corresponding TaqMan assay were run in duplicate and performed on StepOnePlus real-time PCR machine (Applied Biosystems). The cycling conditions comprised 10 min polymerase activation at 95°C and 40 cycles at 95°C for 15 s and 60°C for 1 min. Relative quantification was normalized against the house keeping gene PPIA. The Applied Biosystems assays used for the current study are CD200 (Hs01033303_m1) and cyclophilin A (PPIA) (Hs99999904_m1).

### 2.6. Immunofluorescence

ADHLSCs from 3 different donors at passage 6 grown on glass labtek were fixed with 4% paraformaldehyde (Sigma) for 15 min at room temperature. Nonspecific immunostaining was prevented by 30 min incubation in PBS containing 3% (*w*/*v*) bovine serum albumin (BSA). Cells were incubated for 1 hour with polyclonal goat anti-CD200 primary antibody (AF2724 R&D systems) at room temperature. After washing with PBS, ADHLSCs were incubated for 30 min with donkey anti-goat secondary antibody (A11055, Life Technologies) at room temperature. Nuclei were stained for 5 min with DAPI (Life Technologies). Slides were mounted in fluoromount medium (Sigma). Fluorescence was assessed using Imager A1 fluorescent microscope (Carl Zeiss), and digital images were acquired using AxioVision Software.

### 2.7. Western Blot Analysis

Total protein lysates were obtained by dissolving ADHLSC pellets in RIPA buffer [50 mM Tris base, pH 8.0, 150 mM NaCl, 1% Triton X-100 0.5% sodium deoxycholate, 0.1% SDS, with protein inhibitors cocktail without EDTA (Roche)]. Protein samples were sonicated shortly and incubated for 30 minutes at 4°C prior to sample clarification by centrifugation (15 min maximum speed). Subsequently, sample supernatants were collected and a total protein quantification was performed (BCA quantification kit, Thermofisher). Twenty *μ*g of total protein extracts were dissolved in loading buffer [Tris-HCl (pH 6.8), glycerol, SDS, DTT, and bromophenol blue], denatured at 95°C for 5 minutes and loaded on a 10% Tris-glycine SDS-PAGE gel for protein separation and transferred overnight at 4°C onto PVDF membranes. Membranes were incubated with 5% BSA blocking solution for 90 min at room temperature. CD200 antibody [0.1 *μ*g/ml] was incubated 90 min at room temperature, and the membranes were thoroughly washed 3× with PBS-T, incubated with fluorescently labelled secondary antibody (Biotium) for 40 min at room temperature, and detected by Li-cor scanner (Odyssey). Quantification analysis was performed by Image Studio Lite Software (Odyssey).

### 2.8. Construction of CD200 Protein-Protein Interaction (PPI) Network

We have used STRING database v10 (Search Tool for the Retrieval of Interacting Genes, available at http://string-db.org) [6] with CD200 as seed for construction of the protein-protein interaction (PPI) network.

### 2.9. Statistical Analyses

Results are expressed as mean ± standard error of the mean (SEM). Analyses were done in the GraphPad Prism software program (San Diego, California, USA). Statistical differences were determined by Student's *t*-test for two groups' comparison or by one-way ANOVA followed by the Tukey post hoc test for multiple comparisons between more than two groups. Differences were considered significant when *p* values are ^∗^*p* < 0.05, ^∗∗^*p* < 0.01, and ^∗∗∗^*p* < 0.001.

## 3. Results

### 3.1. Flow Cytometry Characterization of ADHLSCs' Immunological Profile after Hepatogenic Differentiation and Inflammation Priming

**Figure 1:**
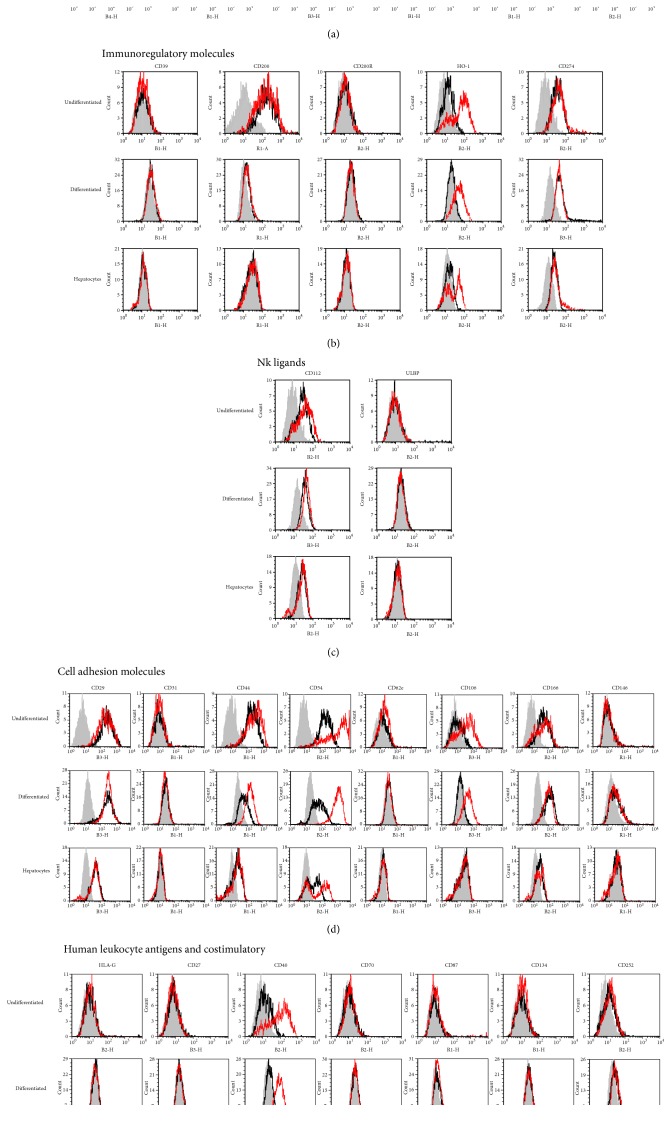
Characterization of the immunoprofile of ADHLSCs and human hepatocytes. Representative FACS histograms indicate the immunoprofiling of different liver-derived cells as determined and analyzed by flow cytometry. The expression of a panel of immunological markers was evaluated in ADHLSCs before and after hepatogenic differentiation and in comparison to hepatocytes being used as standard reference. These liver-derived cells were investigated under constitutive (black curve) and inflammatory-priming conditions (red curve). The grey curve represents the antibody control. The corresponding monoclonal antibodies used for establishing this immunoprofile are listed in Table 1. The data are also presented and listed in Table 2 as the mean ± SEM percentage of each marker expression. (a) Endothelial, stromal, and embryonic markers (CD34, CD45, CD90, CD95, CD210, and CD271). (b) Immunoregulatory molecules (CD39, CD200, CD200R, CD274, and HO-1). (c) Natural killer ligands (CD112, CD155, and ULBP3). (d) Cell adhesion molecules (CD29, CD31, CD44, CD54, CD62e, CD106, CD166, and CD146). (e) Human leukocyte antigens (HLA-G) and costimulatory molecules (CD27, CD40, CD70, CD86, CD134, and CD252).

Flow cytometry analysis was initially carried out to determine the immunologic profile of ADHLSCs under low proliferation conditions and to characterize the impact of hepatogenic differentiation. The quality of hepatogenic differentiation is systematically evaluated at the morphological, expression, and function levels (see Supplementary Figure 1 available online at https://doi.org/10.1155/2017/2679518). The influence of inflammation priming on such profile was also appreciated. Hepatocytes previously isolated from the same donors as ADHLSC were used for comparison as the golden standard reference for all these experimental conditions. The representative FACS histograms (Figure 1) and the related immunopositivity percentages (Table 2) reveal that ADHLSC express a plethora of molecules belonging to several distinct immunological families. Such independent analysis confirmed the mesenchymal profile of ADHLSC and its maintenance in low proliferating and differentiation conditions. Indeed, CD90 was highly and comparably expressed in undifferentiated ADHLSCs (82 ± 3,24%), inflammation-primed undifferentiated ADHLSCs (84 ± 2,52%), differentiated ADHLSC (75,20 ± 7,80%), and inflammation-primed hepatic differentiated ADHLSCs (78,50 ± 8,36%). CD105 expression level exhibited by undifferentiated ADHLSCs (31,83 ± 3,63%) was not significantly changed following inflammation priming (26 ± 2,63%) or after differentiation (29,45 ± 3,46%). Inflammation priming did not significantly impact its expression in differentiated ADHLSCs (28,66 ± 3,47%). Endothelial CD34 and embryonic SSEA4 markers were minimally expressed in the three tested cell types (undifferentiated and differentiated ADHLSCs as well as hepatocytes) without being influenced by inflammation priming.

Comparably to their minimal expression profiles observed in hepatocytes, the cell surface receptors CD45, CD95, CD184, CD200R, CD210, CD229, and CD271 showed a very weak, if any, expression in undifferentiated as well as hepatic-differentiated ADHLSCs. Inflammation priming of the different cell types did not substantially alter the expression level of any of those receptors.

HLA antigens and costimulatory molecules profiling was performed to assess the immunogenicity state of liver-derived cells. As noticed for hepatocytes, both undifferentiated and differentiated ADHLSCs remained nonimmunogenic as the expression of neither HLA-DR and HLA-G nor costimulatory molecules (CD27, CD40, CD70, CD80, CD86, CD134, CD154, and CD252) was significantly induced. On the other hand, and subsequently to inflammation priming, the CD40 expression levels were dramatically increased in both undifferentiated ADHLSCs (from 8,33 ± 0,88 to 70 ± 5,70%) and differentiated ADHLSCs (from 9,33 ± 0,84 to 68,6 ± 4,84) similarly to hepatocytes (from 3,5 ± 0,65 to 72,8 ± 5,22%). HLA-ABC showed high and similar expressions among all the cell groups (undifferentiated ADHLSC (92,83 ± 2,61 versus 93,9 ± 2,88% after inflammation priming), differentiated ADHLSC (96,16 ± 1,47 versus 98,2 ± 0,34 after inflammation priming), and hepatocytes (89 ± 2,97% versus 88 ± 5,02 after inflammation priming).

Profiling of cell adhesion molecules was also evaluated for CD29, CD44, CD49e, CD54, CD58, CD62, CD102, CD106, CD146, and CD166 markers. All exhibited some similarities between undifferentiated ADHLSCs, differentiated ADHLSCs, and hepatocytes. For instance, CD29 and CD166 were highly expressed whilst CD58, CD62, CD102, and CD146 were weakly expressed in all analyzed cell types without striking alterations following both hepatogenic differentiation and inflammation priming. On the other hand, differences were also detected. The CD44 marker was much more expressed in undifferentiated ADHLSC (92,83 ± 1,64%) as compared to differentiated cells (59,50 ± 6,23%) and hepatocytes (57,43 ± 2,40%). Furthermore, CD44 expression was increased following inflammation priming in only differentiated ADHLSC (89,60 ± 3,89%) in contrast to that in undifferentiated cells (93,60 ± 2,26%) and hepatocytes (53,75 ± 4,53%). CD49e expression, part of the fibronectin receptor, was higher in undifferentiated (82 ± 5,43%) as well as in differentiated ADHLSCs (92,98 ± 3,45%) as compared to that in hepatocytes (38,75 ± 7,22%). However, inflammation priming did not exert any striking effect on this integrin expression level in all analyzed cell types. CD54 or intercellular adhesion molecule-1 (ICAM-1) expression was comparable in undifferentiated ADHLSCs (69 ± 5,53%), differentiated ADHLSCs (64,20 ± 5,85%), and hepatocytes (63,25 ± 9%). Inflammation priming of each of those cell types showed a similar elevated CD54 expression in all analyzed cell types (97,80 ± 0,67%, 88,80 ± 4,92%, and 85,50 ± 5,90%, resp.). Although minimally expressed in hepatocytes and differentiated ADHLSC (0,43 ± 0,02% and 1,09 ± 0,82%, respectively) and weak in undifferentiated cells (8,08 ± 1,58%), CD106 expression was substantially induced following inflammation priming of either undifferentiated (48,75 ± 5,54%) or differentiated ADHLSCs (49,50 ± 5,10%) but to a lesser extent in hepatocytes (7,75 ± 4,70%).

As NK cells play a critical role in liver pathology, we determined the expression of the major activating ligands of NK cytolytic activity. The expression pattern of the NK ligands CD112, CD155, and ULBP-3 showed no significant alterations following hepatogenic differentiation or inflammation priming. For instance, the high and comparable CD112 and CD155 expression levels exhibited in undifferentiated ADHLSCs (50 ± 5,82% for CD112; 71,60 ± 4,32% for CD155) and differentiated ADHLSC (46,08 ± 3,35 for CD112; 72,80 ± 4,41 for CD155) were not modulated after inflammation priming of undifferentiated ADHLSCs (58,75 ± 4,30% for CD112; 70,50 ± 2,63% for CD155) as well as differentiated ADHLSCs (43,60 ± 5,24% for CD112; 74 ± 6,55% for CD155). This maintained expression was also noticed in hepatocytes (23,5 ± 1,55% for CD112; 33,75 ± 2,43% for CD155) versus inflammation-primed hepatocytes (25,55 ± 1,93% for CD112; 37,25 ± 3,01% for CD155). On the other hand, ULBP-3 was minimally expressed in all these three cell types without being influenced by inflammation.

Immune regulation and its underlying mechanisms are an important component of liver immunity. The immune-regulatory molecules CD39 and CD274 were constitutively negative in all cell types whereas only CD274 was strongly upregulated after inflammation priming of undifferentiated ADHLSCs (82,40 ± 5,94%), differentiated ADHLSC (64,80 ± 2,26%), and hepatocytes (66,25 ± 2,98%). CD73 and HO-1 molecules exhibited a high constitutive expression level in undifferentiated ADHLSCs (88,8 ± 3,99% for CD73; 65,83 ± 2,21% for HO-1) which remains unchangeable both after hepatogenic differentiation (71,8 ± 6,72% for CD73; 61,83 ± 4,78% for HO-1) and after inflammation priming (90 ± 2,33% for CD73; 66,25 ± 3,10% for HO-1). On the other hand, although HO-1 was substantially and similarly expressed in hepatocytes (32,55 ± 5,10%) and inflammation-primed hepatocytes (35,66 ± 2,02%), CD73 showed only minimal expression under these conditions.

Intriguingly, CD200 being highly expressed in undifferentiated ADHLSCs (69,20 ± 2,96%) was dramatically downregulated following hepatogenic differentiation (2,43 ± 1,19%). Similarly, its expression was very low in mature hepatocytes (0,14 ± 0,08%). In all these experimental cell groups, inflammation priming has any significant impact on CD200 protein expression level.

### 3.2. Modulation of CD200 Expression following Hepatogenic Differentiation

**Figure 2:**
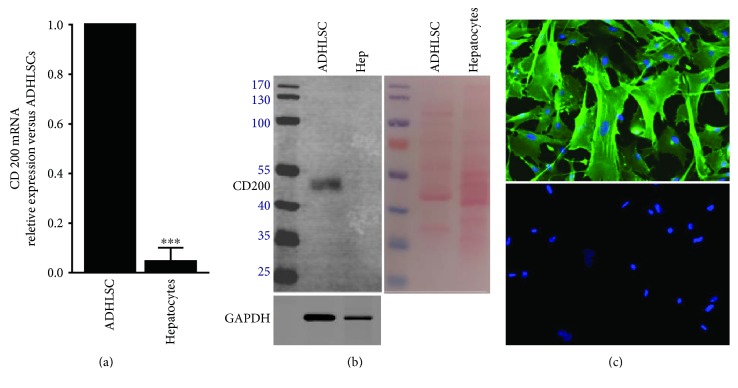
CD200 expression in naïve ADHLSC and mature nonplated hepatocytes. (a) Total RNA extracted from both naïve ADHLSCs and nonplated hepatocytes was retrotranscribed, the cDNA-synthesized and *PPIA*-normalized *CD200* mRNA levels were evaluated by RT-qPCR. A very significant low level of this marker is noticed in mature hepatocytes (*n* = 4) (^∗∗∗^*p* < 0.001 versus naïve ADHLSC, unpaired *t*-test). (b) Cell extracts were prepared and immunoblotted with anti-CD200 and anti-GAPDH antibodies as described in Materials and Methods. Low CD200 protein expression in mature hepatocytes (*n* = 3) is correlated to mRNA expression as compared to ADHLSC. Ponceau S staining shows the quality and the quantity of loading. (c) Immunofluorescence on fixed naïve ADHLSCs was also performed to appreciate the CD200 protein expression. CD200-positive immunoreactivity was significantly confirmed. The lower picture shows that no immunofluorescence signal was observed when cells were incubated with only secondary antibody. Pictures from 3 different donors were taken at a magnification of 200x.

Firstly, quantitative real-time PCR (RT-qPCR) was carried out to estimate the CD200 mRNA level in naïve ADHLSC (expansion culture conditions) and hepatocytes. In agreement with flow cytometry results, RT-qPCR analysis revealed that CD200 transcript expression was extremely low in hepatocytes (Figure 2(a)). This observation is also confirmed by using western blotting (Figure 2(b)). CD200 protein expression in ADHLSC was also confirmed using immunofluorescence and is homogenously distributed at the membrane level (Figure 2(c)).

**Figure 3:**
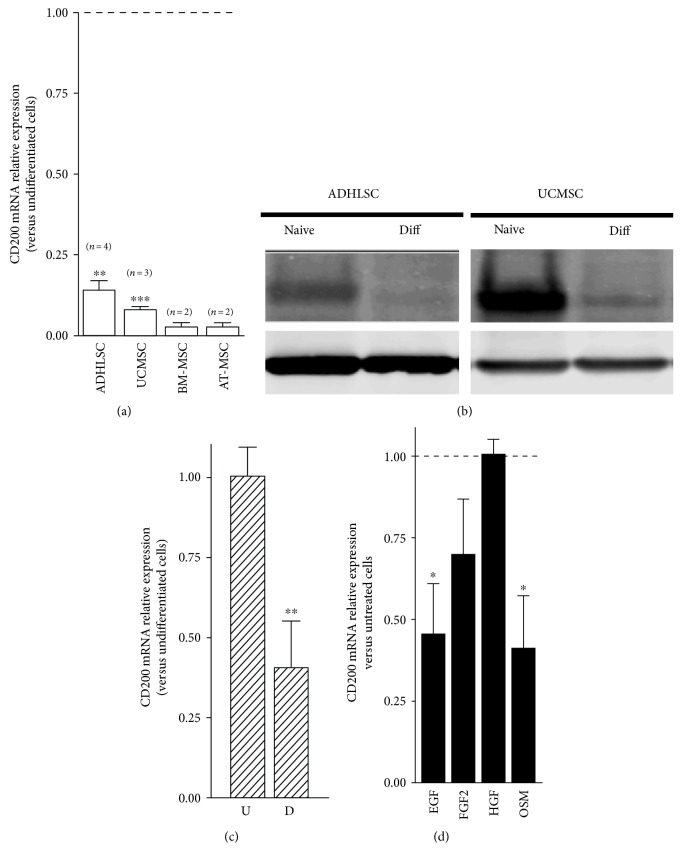
Downregulation of CD200 expression after in vitro hepatogenic differentiation of ADHLSC. ADHLSCs were submitted to an in vitro hepatogenic differentiation protocol and processed for CD200 expression analysis. (a) Total RNA extracted from both undifferentiated and differentiated MSCs (the umbilical cord MSC (UCMSC), bone marrow MSC (BM-MSC), and adipose tissue MSC (AT-MSC)) was retrotranscribed, the cDNA synthesized, and RT-qPCR applied for CD200 gene marker. Hepatogenic differentiation was correlated with a strong downregulation of this marker. (b) Total proteins extracted from differentiated and undifferentiated ADHLSCs and UCMSC were analyzed using western blotting. Hepatogenic differentiation was associated with a significant decrease of CD200 protein expression. (c) Downregulation of CD200 mRNA occurs early at the first step of the hepatogenic differentiation protocol as demonstrated using RT-qPCR. (d) Downregulation of CD200 expression in differentiated ADHLSCs occurs under EGF and oncostatin M treatment. ADHLSCs were treated with one or the other growth factor/cytokine used in the hepatogenic differentiation protocol (epidermal growth factor (EGF), basic fibroblast growth factor (bFGF), hepatocyte growth factor (HGF), or oncostatin M (OSM)). Cells were treated for 3 days using the same basal medium as for differentiation experiments and under serum-free conditions. Total RNA for each group was extracted and retrotranscribed for CD200 mRNA analysis using RT-qPCR. Data shown represent the mean ± SEM of three different experiments as indicated in the graph (^∗^*p* < 0.05; ^∗∗^*p* < 0.01 versus corresponding untreated cells, unpaired *t*-test).

Upon hepatogenic differentiation, we demonstrated that the CD200 mRNA expression level was clearly abolished in differentiated ADHLSCs as compared to undifferentiated cells (Figure 3(a)). Such CD200 downregulation is also observed in MSC from other tissues like the umbilical cord, bone marrow, and adipose tissue and submitted to the same hepatogenic differentiation protocol. Western blot analysis was performed to further assess, at the protein level, the alteration of CD200 expression noticed following hepatogenic differentiation. Consistently with flow cytometry results, CD200 protein expression level exhibited by the undifferentiated ADHLSCs is dramatically diminished after in vitro hepatogenic differentiation. The same results were obtained in both ADHLSCs and UCMSCs (Figure 3(b)).

During hepatogenic differentiation, the downregulation of CD200 looks to be dependent on culture steps. Given that the hepatogenic differentiation protocol is a multistep process, we investigated the kinetic of CD200 downregulation. Our data demonstrated that CD200-decreased mRNA expression happens starting from the first step (Figure 3(c)). Thereafter, we treated ADHLSC separately with each of the growth factors used in the cocktail of differentiation to gain insights regarding the potential signaling events behind the dramatic shutdown of CD200 expression following hepatogenic differentiation. As shown in Figure 3(d), data presented as a relative CD200 mRNA level in treated versus untreated ADHLSC (serum-free conditions) clearly shows that HFG and bFGF exerted no effect and a slightly nonsignificant effect, respectively. Nevertheless, both EGF and OSM treatments resulted in a significant decrease of about half of the CD200 mRNA level. Such diminution is in line with the decreased CD200 mRNA levels observed after the first and the last steps of the hepatogenic differentiation protocol.

**Figure 4:**
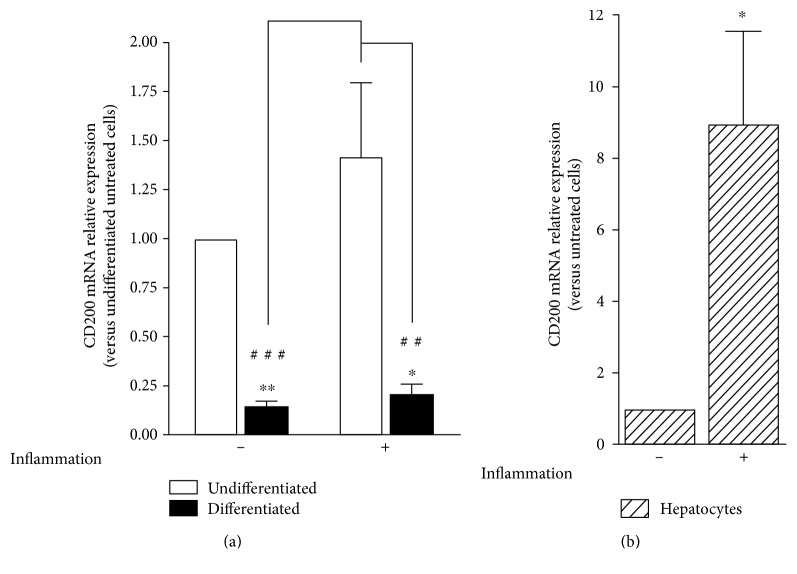
CD200 mRNA expression after inflammation priming. Undifferentiated ADHLSCs, (a) differentiated ADHLSCs as well as (b) hepatocytes were grown under basic or inflammatory-priming conditions. Total RNA was isolated and *PPIA*-normalized *CD200* mRNA levels were quantified by RT-qPCR. Data shown represent the mean ± SEM of three different experiments (^∗^*p* < 0.05; ^∗∗^*p* < 0.01 versus corresponding untreated cells, unpaired *t*-test; ^##^*p* < 0.01; ^###^*p* < 0.001 versus corresponding treated undifferentiated cells, one-way ANOVA followed by the Tukey post hoc test).

Finally, we checked if CD200 mRNA expression was also modulated after inflammation priming. As shown in Figure 4, this parameter was investigated in undifferentiated and differentiated ADHLSCs as well as in hepatocytes. Whilst no effect was observed in ADHLSCs (Figure 4(a)), inflammation priming of hepatocytes strongly induced the CD200 mRNA level (Figure 4(b)).

**Figure 5:**
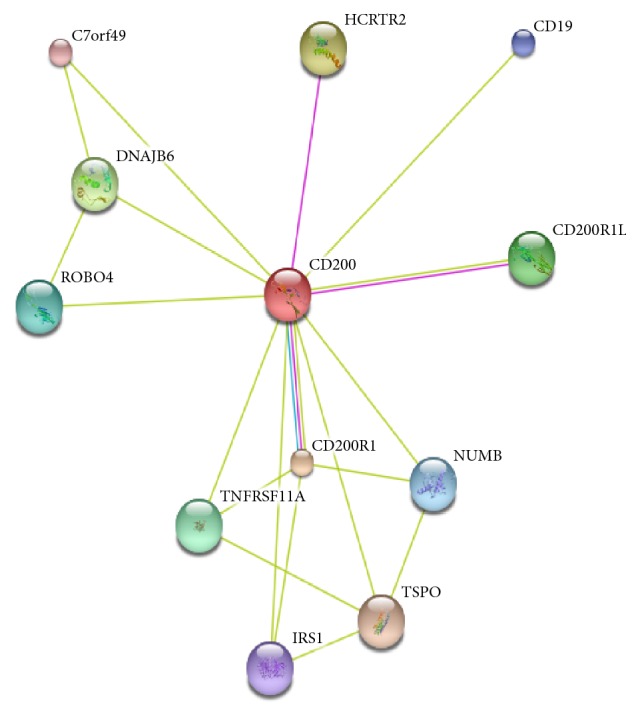
STRING database-generated protein interaction network generated using CD200 protein name as query. The blue and pink colours correspond to known interactions from curated and experimental work, respectively, whilst the light-green colour corresponds to interaction prediction based on text mining.

The functional network of CD200 and its interacting proteins was analyzed to better highlight which pathways might be altered during hepatogenic differentiation. Due to its great immunological importance, CD200 protein network was analyzed by using STRING version10 database. This analysis was selected for its many advantages among which extensive collection of precomputed interaction data derived from various sources, such as, high-throughput experimental data, literature data, and computational predictions. This tool was used to query, retrieve, and analyze the CD200 protein interaction network with the interactions restricted to those available for *Homo sapiens*. Using CD200 as a query, and choosing the prediction methods to include neighborhood, gene fusion, co-occurrence, coexpression, experiments, databases, and text mining, a network of eleven interacting proteins was constructed (Figure 5). This interaction network is visualized in the form of a graph with the protein molecules forming the nodes of the graph and the interactions forming the edges. Among those eleven predicted interacting proteins, four were already confirmed to interact with CD200 among which three (CD200R1, HCRTR2, and CD200R1L) were experimentally determined, and one (CD200R1) was determined from a curated database whilst the remaining seven were predicted based on text mining and were verified using PubMed literature database. Indeed, CD200R1 and CD200R1L (CD200R2) are receptors for CD200 whilst HCRTR2 is a hypocretin (orexin) receptor 2 implicated in neuropeptide signaling pathway [7]. Orexins and orexin receptors, a family of hypothalamic neuropeptides and G protein-coupled receptors are involved in the regulation of feeding behavior [7] in addition to their ability to trigger phospholipase C signaling via activating Ca2^+^ influx [8].

## 4. Discussion

By maintaining vital metabolic homeostasis, good functioning of the liver is essential for survival. Due to its high ability to regenerate and to its continuous extrahepatic cellular supply, cell therapy is currently appreciating the potential of exogenous cell suspensions in replenishing the liver and correcting its functional and/or structural defects. Extensive preclinical work has been conducted to study the mechanistic pathways that govern genesis of the major liver cells, hepatocytes. Nowadays, different stem cells are being investigated to hopefully increase the diversity of cell pools and availability of cell-based therapy products for patients who can no longer regenerate their altered hepatic tissue [9]. In addition to adult bone marrow-derived and embryonic stem cells that have been well described for their potential to transdifferentiate into mature hepatocyte-like cells [9], ADHLSCs have recently emerged as a novel progenitor reservoir of hepatocytes [2, 4, 10]. ADHLSC in vitro and in vivo hepatocytic differentiation potential [2, 4] and hypoimmunogenic profile [5] support their usefulness for liver regeneration [2–5, 10].

Acceptance of engrafted ADHLSCs could be a result of the surrounding inflammatory condition sensing as well as the modulation of the host immune responses like to what has been reported for other MSCs [11, 12]. Based on the interesting expression and secretion profiles of naïve ADHLSC [3], the current study aimed at appreciating the impact of hepatogenic differentiation and inflammation on those features. In agreement with our recent observations [5], we confirmed that ADHLSCs are negative for CD34 and SSEA-4 but positive for CD90 and CD105 with no influence of inflammation and differentiation on that expression profile. Of note, CD90, which is absent in mature hepatocytes, remains expressed in differentiated ADHLSCs. This constitutive CD90 expression could be advantageous since decreased expression of CD90 has been reported to impair MSC immunosuppressive capacity [13].

Consistent with the previously reported ADHLSCs' hypoimmunogenicity [2, 5], we observed that, regardless of inflammation priming, undifferentiated and differentiated ADHLSCs as well as hepatocytes are negative for HLA-DR, HLA-G, and the costimulatory molecules CD27, CD70, CD80, CD86, CD134, CD154, and CD252 but positive for HLA-ABC molecules. The absence of CD134 and CD252 in differentiated ADHLSCs is of special interest because of the ability of these molecules to impair the suppressive action of regulatory T cells and thus to trigger host immune responses [14]. The increased CD40 expression, following inflammation priming, may thus not be sufficient to activate T cells since such activation involves other costimulatory molecules that appeared to be negative in our case [15].

Furthermore, our data showed that undifferentiated and differentiated ADHLSCs as well as hepatocytes are all negative for the surface receptors CD45 (a marker used to identify and isolate human hepatic progenitor cells) [16], CD95 (a marker that triggers apoptosis in liver cells) [17], CD184 (involved in repairing liver injury upon triggering MSCs to migrate, transdifferentiate, and fuse with hepatocytes) [18], CD200R (involved in mediating anti-inflammatory hepatic responses) [19], CD210 (playing important anti-inflammatory and immunosuppressive roles in liver) [20], CD229 (involved in the control of hepatocyte proliferation) [21], and CD271 (a marker of human hepatic stellate cells) [22]. This expression profile is not influenced after inflammation priming.

Regarding cell adhesion molecules (CAMs), undifferentiated ADHLSCs, differentiated ADHLSCs, and hepatocytes are negative for CD31 (with anti-inflammatory properties in the liver) [23], CD58 (proposed to augment cell mediated immunity against hepatitis B virus and thus leading to hepatocyte destruction) [24], CD62e, CD102 (involved in recruitment of lymphocytes to the liver) [25], and CD146 (although poorly characterized but described to be involved in angiogenesis) [26]. However, all cells are positive to CD29 (essential for hepatocyte survival) [27], CD49e, and CD166 (playing an antiapoptotic role for liver cells) [28] regardless of presence or absence of inflammatory signals. Our observations for CD102, CD146, and CD166 expression levels in undifferentiated ADHLSCs are in line with our previous observations [5]. CD44 (involved in regulating liver inflammation) [29] and CD54 (involved in the recruitment of hepatic-natural killer cells in the liver) [30] exhibit high levels in undifferentiated and differentiated ADHLSCs as well as hepatocytes. Inflammatory signals reinforce CD54 expression in these different cells and increase CD44 expression only in differentiated ADHLSCs. Remarkably, the minimal CD106 expression (involved in enabling neutrophil migration within the liver) [31] exhibited by both undifferentiated and differentiated ADHLSCs was strongly induced by inflammatory signals to levels higher than those exhibited by inflammation-primed hepatocytes. These observations indicate that the different examined CAMs are heterogeneously expressed in the different tested cell types and exhibit different sensitivities to inflammation.

As a key component of the liver's innate immunity, NK cells are primarily involved in host defence but also have regulatory effects during their interactions with other types of liver cells [32]. Regardless of inflammation priming, both differentiated ADHLSCs and hepatocytes were positive for CD112 and CD155 but negative for ULBP-3, in a manner comparable to that exhibited by undifferentiated ADHLSCs. NK cells' lytic activity could be triggered upon interaction of CD112 or CD155 with the same NK-activating receptor DNAM-1 [33] or ULBP-3 with NKG2D receptor [34]. Although the absence of ULBP-3 seems advantageous, the presence of CD112 and CD155 might be risky in terms of allowing NK-mediated lysis of those liver-derived cells.

Given that ADHLSCs could exhibit immunomodulatory properties, it was then important to evaluate the expression and modulation of several relevant immune regulatory molecules. Undifferentiated and differentiated ADHLSCs were positive for CD73 and HO-1 but negative for CD39, regardless of inflammation priming. Interestingly, although initially negative for CD274, inflammation strikingly increased its expression on all the studied cells. CD274 expression is known to be upregulated in response to proinflammatory cytokines where it exerts an inhibitory role on the costimulatory pathway of T cells [35]. CD274 upregulation could then compensate the effect of upregulated CD40 expression observed after inflammation priming, thus inhibiting T cell activation.

A major output of this study is the downregulation of CD200 expression that takes place during hepatogenic differentiation. This impaired CD200 expression in differentiated ADHLSCs is consistent with the absence of CD200 expression in hepatocytes. CD200 is a transmembrane surface glycoprotein which, upon engagement with its receptor (CD200R), delivers inhibitory signals leading to immunosuppression, inhibition of inflammation, and tolerance to allografts [33]. Given the important inflammation-inhibitory potential of CD200, the observed loss of its expression in differentiated ADHLSCs could limit their anti-inflammatory capacity, thus imposing undesired consequences during the application of ADHLSC-based liver therapy. CD200 is widely expressed in many cell types, among which are extrahepatic MSCs. This expression is uneven and can be modulated depending on the tissue origin and growth conditions. For instance, CD200 expression is not equal in the bone marrow, umbilical cord blood, Wharton's jelly, and adipose tissue-derived MSCs [36, 37]. Of great importance, hepatic progenitor cells (HPCs) isolated from the adult human liver are highly similar to cultured primary hepatocytes in their transcriptional profiles where both cell types are negative for CD200 expression [16]. Expression of several MSC markers including CD73, CD146, and CD200 is downregulated after adipogenic and osteogenic differentiation [38] whilst only CD200 expression is completely abolished following chondrogenic differentiation [38]. Identifying specific markers to evaluate the multilineage differentiation potential of stem cells is thus highly important for the improvement of stem-cell-based therapies. CD105 expression was demonstrated to be significantly downregulated following adipogenic, chondrogenic, and osteogenic differentiation of umbilical cord blood MSCs [39]. In addition, to be used as a specific marker for a determined cell population or for a defined cell differentiation status, the observations made in this study might suggest CD200 as a marker for the immunomodulatory potential of ADHLSCs.

Protein-protein interaction (PPI) network revealed that several proteins may interact with CD200. These proteins may be expressed by different liver-derived cells and may also have implications in various cell biology features such as for roundabout, axon guidance receptor, homolog 4 (Robo4) in cell migration and angiogenesis during endothelial inflammatory responses [40, 41], and insulin receptor substrate 1 (IRS-1) that may mediate the control of various cellular processes related to insulin and insulin-like growth factor-1 (IGF-1) receptors [42]. Following the engagement of CD200 with CD200 receptor 1, the tumor necrosis factor receptor superfamily member 11a (TNFRSF11A) can interact with the TNF receptor-associated factors (TRAFs) [43] which modulate the immune and inflammatory responses particularly during the interaction between T cells and dendritic cells [43]. The potential interaction of CD200 with translocator protein (TSPO) could have important consequences for ADHLSC therapeutic applications because of its involvement in the regulation of cellular metabolic energy. TSPO, a mitochondrial outer membrane protein that belongs to a family of tryptophan-rich sensory, modulates critical biological functions, such as cellular bioenergetics and metabolism, immunomodulation, and apoptosis [44]. The juxtaposition of TSPO at the cytosolic/mitochondrial interface and the existence of endogenous ligands that are regulated by metabolism suggest that TSPO functions to adapt mitochondrial to cellular metabolism [45]. Indeed, TSPO has been shown to affect mitochondrial energy homeostasis through modulation of fatty acid oxidation (FAO). Such function positively correlates with high levels of TSPO expression as observed in cell types active in lipid storage/metabolism [46]. From a pharmacological perspective, the specific upregulation of TSPO in inflammatory and injury conditions makes TSPO an interesting, druggable target of mitochondrial metabolism [47].

The potential interactions of CD200 with Numb, a cell fate determinant with a critical role in the regulation of pluripotency and stem cell division [48], are of importance. Indeed, Numb phosphorylation by pluripotency-associated transcription factor NANOG and subsequent p53 degradation drive self-renewability and proliferation of tumor-initiating cells, which results in higher liver oncogenesis [49]. Second, Numb inhibits the Notch pathway, a crucial regulator of stem cell behavior [50]. Several mechanisms of actions are proposed [50] including the ubiquitination of Notch1 receptor and the subsequent degradation of the Notch intracellular domain following receptor activation [51] and the inhibition of Notch endosomal trafficking and recycling [52]. In the context of ADHLSC cell therapy, CD200 and Numb interactions could play a crucial role to maintain the proper homeostasis of both undifferentiated and differentiated cells.

In conclusion, we suggest that the modulation of the CD200 pathway might be involved in the control of the differentiated ADHLSC immunosuppressive function. Whilst requiring further investigations, the current data are helpful for tackling the interest of deeply exploring the immune biology of liver cells to transplant for an efficient management of liver diseases by cell therapy-based approaches. Thanks to their ability to functionally differentiate into hepatocyte-like cells and to maintain the major immunological profile of mature hepatocytes, differentiated ADHLSCs might support their development for liver cell therapy.

## Supplementary Material

ADHLSCs were sequentially incubated with specific growth factors/cytokines and processed for the evaluation of the hepatogenic differentiation quality. A) Differentiated ADHLSC display significant morphological changes with polygonal epithelial-like shape. Pictures were taken at magnification of 200x. Presented data are representative of at least three different experiments. B) RT-PCR analysis of hepatocyte specific gene expression profile of differentiated (Diff) compared to undifferentiated ADHLSCs (Und) confirms a positive correlation with the morphological changes. Data shown are agarose gel electrophoresis of amplification products corresponding to hepatic markers: MRP2, multidrug resisting protein-2; TDO, tryptophan 2,3-dioxygenase; CYP3A4, cytochrome P450, family 3, subfamily A, polypeptide 4; GAPDH, glyceraldehyde-3-phosphate dehydrogenase is used as house-keeping control. Presented data are representative of at least three different experiments. C) Forty μg of total protein extracted from differentiated ADHLSCs and isolated hepatocytes were analyzed using western blotting. Hepatogenic differentiation was supported, by demonstrating the expression of CYP3A4 and hepatocyte nuclear factor-4 alpha (HNF4a) proteins in differentiated ADHLSC (Diff) as compared to hepatocytes (Hep). D) After the hepatogenic differentiation process, undifferentiated (U) and differentiated (D) ADHLSCs were recovered for CYP3A4 activity analysis using P450-GloTM assay a Victor3 luminometer (PerkinElmer). Data shown are the mean ± SEM of three independent experiments (T-test ∗∗∗ p< 0.001 vs undifferentiated ADHLSC)

## Figures and Tables

**Table 1:** Conjugated monoclonal antibodies used for flow cytometry analysis.

Primary antibody	Order ID	Species	Dilution	Source
*Endothelial, stromal, and embryonic markers*				
Anti-CD34-PC5	555823	Mouse	1/20	BD
Anti-CD90-PE	FAB2067P	Mouse	1/20	R&D
Anti-CD105-FITC	326040	Mouse	1/20	AC
Anti-SSEA4-PE	FAB1435P	Mouse	1/20	R&D

*Receptors*				
Anti-CD45-PC7	557748	Mouse	1/20	BD
Anti-CD95-FITC	130092415	Mouse	1/20	MB
Anti-CD184-PE	555974	Mouse	1/20	BD
Anti-CD200R-PE	329306	Mouse	1/20	BL
Anti-CD210-PE	556013	Rat	1/20	BD
Anti-CD271-PE	120002227	Mouse	1/20	MB
Anti-CD229-PE	326108	Mouse	1/20	BL

*Human leukocyte antigens*				
Anti-HLA-ABC-PE-Cy5	15998342	Mouse	1/20	EB
Anti-HLA-DR-PerCP	347402	Mouse	1/20	BD
Anti-HLA-G-PE	1P292C100	Mouse	1/20	ExBIO

*Costimulatory molecules*				
Anti-CD27-APC-Cy7	302815	Mouse	1/20	BL
Anti-CD40-PE	130094135	Mouse	1/20	MB
Anti-CD70-PE	355104	Mouse	1/20	BL
Anti-CD80-FITC	11080942	Mouse	1/20	EB
Anti-CD86-APC	130094876	Mouse	1/20	MB
Anti-CD134-FITC	350006	Mouse	1/20	BL
Anti-CD154-PE-Cy5	310808	Mouse	1/20	BL
Anti-CD252-PE	326308	Mouse	1/20	BL

*Cell adhesion molecules*				
Anti-CD29-PE-Cy5	559882	Mouse	1/20	BD
Anti-CD31-PE	130092653	Mouse	1/20	MB
Anti-CD44-FITC	130095195	Mouse	1/20	MB
Anti-CD49e-PE	555617	Mouse	1/20	BD
Anti-CD54-PE	555511	Mouse	1/20	BD
Anti-CD58-FITC	555920	Mouse	1/20	BD
Anti-CD62e-fluorescein	BBA21	Mouse	1/20	R&D
Anti-CD102-FITC	328507	Mouse	1/20	BL
Anti-CD106-PE-Cy5	551148	Mouse	1/20	BD
Anti-CD146-PC5	A22364	Mouse	1/20	BC
Anti-CD166-PE	559263	Mouse	1/20	BD

*Immunoregulatory molecules*				
Anti-CD39-FITC	328205	Mouse	1/20	BL
Anti-CD73-PE	344003	Mouse	1/20	BD
Anti-CD200-APC	329208	Mouse	1/20	BL
Anti-CD274-PE	557924	Mouse	1/20	BD
Anti-HO-1-PE	ADI-OSA-111	Mouse	1/20	ELS

*NK ligands*				
Anti-CD112-PE	337410	Mouse	1/20	BL
Anti-CD155-PE	337508	Mouse	1/20	BL
Anti-ULBP-3-PE	FAB1517P	Mouse	1/20	R&D

**Table 2:** Expression levels of different molecules present on undifferentiated and differentiated ADHLSC as well as hepatocytes being cultivated in the presence or absence of inflammation cocktail. The data are presented as the mean ± SEM (standard error of the mean) percentage of each marker expression.

	ADHLSCs				Hepatocytes	
	Undifferentiated		Differentiated			
	Constitutive	Priming	Constitutive	Priming	Constitutive	Priming
*Endothelial, stromal, and embryonic markers*						
CD34	0,96 ± 0,28	0,54 ± 0,17	0,37 ± 0,15	0,9 ± 0,28	3,5 ± 0,64	1,33 ± 0,92
CD90	82 ± 3,24	84 ± 2,52	75,20 ± 7,80	78,50 ± 8,36	0,32 ± 0,15	0,34 ± 0,22
CD105	31,83 ± 3,63	26 ± 2,63	29,45 ± 3,46	28,66 ± 3,47	0,40 ± 0,20	0,1 ± 0,04
SSEA4	1,4 ± 0,38	2,52 ± 0,40	1,62 ± 0,31	1,98 ± 0,50	0,57 ± 0,19	1,96 ± 0,52

*Receptors*						
CD45	0,78 ± 0,23	1,29 ± 0,46	1,38 ± 0,60	0,71 ± 0,18	0,12 ± 0,08	2,08 ± 1,69
CD95	2,45 ± 0,58	6,80 ± 1,94	3,25 ± 1,14	7,34 ± 1,92	0,20 ± 0,17	2 ± 0,86
CD184	1,53 ± 0,55	2,06 ± 0,68	1,13 ± 0,18	1,06 ± 0,20	0,49 ± 0,23	0,73 ± 0,15
CD200R	1,02 ± 0,27	1,23 ± 0,12	0,79 ± 0,08	1,13 ± 0,33	0,32 ± 0,08	0,69 ± 0,29
CD210	0,73 ± 0,19	1,6 ± 0,10	1,23 ± 0,13	1,30 ± 0,27	0,30 ± 0,18	0,66 ± 0,24
CD271	2,18 ± 0,79	2,20 ± 0,34	2,33 ± 0,61	2,80 ± 0,60	0,42 ± 0,18	1,36 ± 0,72
CD229	0,66 ± 0,17	0,53 ± 0,13	0,34 ± 0,09	0,47 ± 0,14	0,28 ± 0,08	0,25 ± 0,03

*Human leukocyte antigens*						
HLA-ABC	92,83 ± 2,61	93,9 ± 2,88	96,16 ± 1,47	98,2 ± 0,34	89 ± 2,97	88 ± 5,02
HLA-DR	0,78 ± 0,28	0,66 ± 0,23	0,6 ± 0,33	0,4 ± 0,15	1,5 ± 0,3	1,52 ± 1,49
HLA-G	2,43 ± 1,13	1,62 ± 0,37	1,57 ± 0,28	1,58 ± 0,6	0,29 ± 0,16	0,47 ± 0,13

*Costimulatory molecules*						
CD27	0,27 ± 0,12	0,57 ± 0,20	1,06 ± 0,34	0,3 ± 0,12	0,36 ± 0,1	0,5 ± 0,07
CD40	8,33 ± 0,88	70 ± 5,70	9,33 ± 0,84	68,6 ± 4,84	3,5 ± 0,65	72,8 ± 5,22
CD70	0,84 ± 0,22	0,87 ± 0,27	0,79 ± 0,30	0,57 ± 0,15	0,14 ± 0,06	0,71 ± 0,20
CD80	1,13 ± 0,34	1,66 ± 0,77	1,06 ± 0,60	1,44 ± 0,83	1,43 ± 0,07	1,53 ± 0,05
CD86	0,75 ± 0,18	0,67 ± 0,31	0,76 ± 0,34	1,18 ± 0,44	2,34 ± 1,02	1,05 ± 0,84
CD134	1,56 ± 0,60	1,30 ± 0,75	1,66 ± 0,59	1,58 ± 0,67	0,26 ± 0,10	0,13 ± 0,05
CD154	0,42 ± 0,12	1,03 ± 0,33	2 ± 0,40	1,36 ± 0,33	0,45 ± 0,10	0,25 ± 0,10
CD252	5 ± 1,06	4,65 ± 0,20	3 ± 0,57	4,60 ± 0,71	0,43 ± 0,15	0,52 ± 0,10

*Cell adhesion molecules*						
CD29	91 ± 4,80	89,60 ± 4,94	95 ± 1,38	97 ± 0,65	84 ± 3	89 ± 3,82
CD31	1,2 ± 0,45	1,7 ± 0,64	1,3 ± 0,66	1,6 ± 0,93	0,26 ± 0,11	0,31 ± 0,13
CD44	92,83 ± 1,64	93,60 ± 2,26	59,50 ± 6,23	89,60 ± 3,89	57,43 ± 2,40	53,75 ± 4,53
CD49e	82 ± 5,43	89,75 ± 3,87	92,98 ± 3,45	97,23 ± 0,51	38,75 ± 7,22	34,33 ± 5,29
CD54	69 ± 5,53	97,80 ± 0,67	64,20 ± 5,85	88,80 ± 4,92	63,25 ± 9	85,50 ± 5,90
CD58	3,12 ± 1,17	7,44 ± 2,74	2,68 ± 1,47	3,06 ± 1,42	0,80 ± 0,45	3,50 ± 0,65
CD62e	1,96 ± 0,52	2,4 ± 0,98	1,56 ± 0,57	3,03 ± 1,63	0,2 ± 0,09	0,9 ± 0,42
CD102	1 ± 0,25	0,98 ± 0,56	0,88 ± 0,30	1,40 ± 0,72	0,1 ± 0,04	0,1 ± 0,01
CD106	8,08 ± 1,58	48,75 ± 5,54	1,09 ± 0,82	49,50 ± 5,10	0,43 ± 0,02	7,75 ± 4,70
CD146	4,83 ± 1,51	3,64 ± 0,82	3,16 ± 0,60	4 ± 0,64	0,1 ± 0,033	0,04 ± 0,038
CD166	25,60 ± 1,64	24,4 ± 1,31	26,83 ± 2,03	27,20 ± 3,71	30,93 ± 0,05	31,67 ± 3,50

*Immunoregulatory molecules*						
CD39	1,55 ± 0,54	1,48 ± 0,64	2,68 ± 1,29	2,52 ± 1,26	0,03 ± 0,02	0,09 ± 0,01
CD73	88,8 ± 3,99	90 ± 2,33	71,8 ± 6,72	79,75 ± 5,79	2,01 ± 0,08	1,8 ± 0,52
CD200	69,20 ± 2,96	60 ± 8,05	2,43 ± 1,19	1,86 ± 1,05	0,14 ± 0,08	0,61 ± 0,47
CD274	1,50 ± 0,35	82,40 ± 5,94	0,76 ± 0,13	64,80 ± 2,26	0,60 ± 0,34	66,25 ± 2,98
HO-1	65,83 ± 2,21	66,25 ± 3,10	61,83 ± 4,78	68,60 ± 4,22	32,55 ± 5,10	35,66 ± 2,02

*NK ligands*						
CD112	50 ± 5,82	58,75 ± 4,30	46,08 ± 3,35	43,60 ± 5,24	23,5 ± 1,55	25,55 ± 1,93
CD155	71,60 ± 4,32	70,50 ± 2,63	72,80 ± 4,41	74 ± 6,55	33,75 ± 2,43	37,25 ± 3,01
ULBP-3	1,07 ± 0,26	1,46 ± 0,42	0,72 ± 0,16	1,20 ± 0,52	0,53 ± 0,25	1 ± 0,21

## Abbreviations

ADHLSC: Adult-derived human liver stem/progenitor cells
bFGF: Basic fibroblast growth factor
EGF: Epidermal growth factor
HGF: Hepatocyte growth factor
NK: Natural killer
OSM: Oncostatin M.
