# Supplementary material for: Immunoprofiling of Adult-Derived Human Liver Stem/Progenitor Cells: Impact of Hepatogenic Differentiation and Inflammation
Source: Stem Cells Int. 2017 Apr 11;2017:2679518. doi: 10.1155/2017/2679518 (PMC5405586; doi:10.1155/2017/2679518)
Supplement: Supplementary file 1 — ADHLSCs were sequentially incubated with specific growth factors/cytokines and processed for the evaluation of the hepatogenic differentiation quality. A) Differentiated ADHLSC display significant morphological changes with polygonal epithelial-like shape. Pictures were taken at magnification of 200x. Presented data are representative of at least three different experiments. B) RT-PCR analysis of hepatocyte specific gene expression profile of differentiated (Diff) compared to undifferentiated ADHLSCs (Und) confirms a positive correlation with the morphological changes. Data shown are agarose gel electrophoresis of amplification products corresponding to hepatic markers: MRP2, multidrug resisting protein-2; TDO, tryptophan 2,3-dioxygenase; CYP3A4, cytochrome P450, family 3, subfamily A, polypeptide 4; GAPDH, glyceraldehyde-3-phosphate dehydrogenase is used as house-keeping control. Presented data are representative of at least three different experiments. C) Forty μg of total protein extracted from differentiated ADHLSCs and isolated hepatocytes were analyzed using western blotting. Hepatogenic differentiation was supported, by demonstrating the expression of CYP3A4 and hepatocyte nuclear factor-4 alpha (HNF4a) proteins in differentiated ADHLSC (Diff) as compared to hepatocytes (Hep). D) After the hepatogenic differentiation process, undifferentiated (U) and differentiated (D) ADHLSCs were recovered for CYP3A4 activity analysis using P450-GloTM assay a Victor3 luminometer (PerkinElmer). Data shown are the mean ± SEM of three independent experiments (T-test ∗∗∗ p< 0.001 vs undifferentiated ADHLSC) [file 2679518.f1.pptx]

## Slide 1
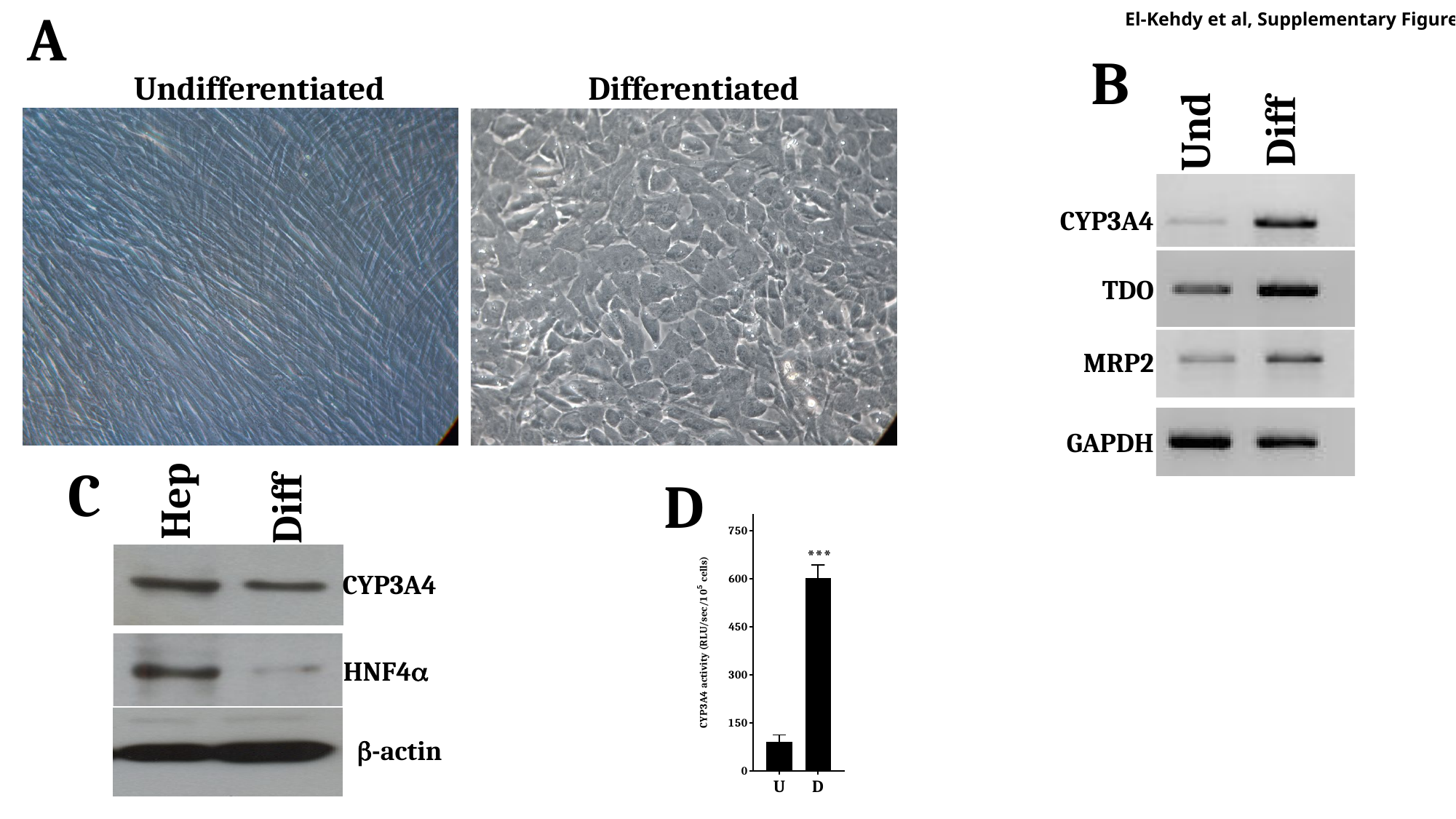

A
El-Kehdy et al, Supplementary Figure 1
B
Undifferentiated Differentiated
Und
Diff
CYP3A4
TDO
MRP2
GAPDH
C
D
Hep
Diff
CYP3A4
HNF4a
b-actin
