# Supplementary material for: Immunoprofiling of Adult-Derived Human Liver Stem/Progenitor Cells: Impact of Hepatogenic Differentiation and Inflammation
Source: Stem Cells Int. 2017 Apr 11;2017:2679518. doi: 10.1155/2017/2679518 (PMC5405586; doi:10.1155/2017/2679518)
Supplement: Supplementary file 2 [file 2679518.f2.docx]

**Legend of the supplementary figure 1**

ADHLSCs were sequentially incubated with specific growth factors/cytokines and processed for the evaluation of the hepatogenic differentiation quality.

***A)*** Differentiated ADHLSC display significant morphological changes with polygonal epithelial-like shape. Pictures were taken at magnification of 200x. Presented data are representative of at least three different experiments.

***B)*** RT-PCR analysis of hepatocyte specific gene expression profile of differentiated (Diff) compared to undifferentiated ADHLSCs (Und) confirms a positive correlation with the morphological changes. Data shown are agarose gel electrophoresis of amplification products corresponding to hepatic markers: MRP2, multidrug resisting protein-2; TDO, tryptophan 2,3-dioxygenase; CYP3A4, cytochrome P450, family 3, subfamily A, polypeptide 4; GAPDH, glyceraldehyde-3-phosphate dehydrogenase is used as house-keeping control. Presented data are representative of at least three different experiments.

***C)*** Forty µg of total protein extracted from differentiated ADHLSCs and isolated hepatocytes were analyzed using western blotting. Hepatogenic differentiation was supported, by demonstrating the expression of CYP3A4 and hepatocyte nuclear factor-4 alpha (HNF4a) proteins in differentiated ADHLSC (Diff) as compared to hepatocytes (Hep).

***D)*** After the hepatogenic differentiation process, undifferentiated (U) and differentiated (D) ADHLSCs were recovered for CYP3A4 activity analysis using P450-GloTM assay a Victor3 luminometer (PerkinElmer). Data shown are the mean ± SEM of three independent experiments (T-test *** *p*< 0.001 vs undifferentiated ADHLSC)

**Materials & Methods used**

***Reverse transcription– polymerase chain reaction***

Total RNA was extracted as previously described [2]. PCR amplifications were performed using polymerase elongase in a final volume of 25 µl and appropriate primers as those described in [2]. Samples were thereafter electrophoresed on a 1 % agarose gel and amplicons were visualized by ethidium bromide staining.

***Immunoblotting***

# The protocol performed is described in Materials & Methods section of the main document. The antibody CYP3A4 (Chemicon, Ref: Ab1254) and HNF4α (LSBio, LS-B6579) were used at dilutions 1/100 and 1/1000 respectively. β-actin was used as internal control for loading (Abcam Ab6276) at concentration 0.1 µg/mL

***CYP3A activity***

CYP3A activity in undifferentiated and differentiated ADHLSC was analyzed using P450-Glo^TM^ assay according to the manufacturer’s instructions and as previously described [2].
